# Lectures on Theory and Practice of Physic

**Published:** 1849

**Authors:** 


### ARTICLE IX.

*Lectures on the Theory and Practice of Physic.* By JOHN BELL, M. D., Member of the Amer. Med. Association &c., &c., and by WILLIAM STOKES, M. D., Lecturer at the Medical School, Park Street, Dublin; Physican to the Meath Hospital, &c., &c. Fourth Edition, revised and enlarged. In two volumes. pp. 1760. Philadelphia: Ed. Barrington and Geo. D. Haswell, 1848. (From the Publishers and for sale by Keenes, Chicago.)

The fourth edition of this excellent standard work, comes to us revised and enlarged by the American contributor. It is with great propriety termed Bell and Stokes' Practice, instead of the title under which it was at first issued, (Stokes and Bell's,) since Dr. Bell has contributed nearly four fifth's of the matter contained in the present edition. The new articles in this edition are on diseases of the eyes, diseases of the blood-vessels, and dropsy. Besides these, the author tells us in his preface that several of the most important subjects, such as "epidemic cholera, diseases of the urinary organs, of the female organs of generation, pulmonary consumption, diseases of the heart, meningitis (simple and tubercular), the exanthemata, and fevers," have been recast.

It is now as complete a system of practice as any extant, and has, for some time been recommended, as a text book, in several of the best medical institutions in the country.

